# Targeting the Tumor Microbiota in Cancer Therapy Basing on Nanomaterials

**DOI:** 10.1002/EXP.20210185

**Published:** 2025-03-24

**Authors:** Yanan Niu, Junya Feng, Jie Ma, Tixian Xiao, Wei Yuan

**Affiliations:** ^1^ State Key Laboratory of Molecular Oncology, National Cancer Center/Cancer Hospital, Chinese Academy of Medical Sciences Peking Union Medical College Beijing P. R. China; ^2^ Department of Biotherapy, Beijing Hospital, National Center of Gerontology, Institute of Geriatric Medicine, Chinese Academy of Medical Sciences Graduate School of Peking Union Medical College Beijing P. R. China; ^3^ Department of Colorectal Surgery National Cancer Center/Cancer Hospital Chinese Academy of Medical Sciences Peking Union Medical College Beijing P. R. China; ^4^ AdventHealth Medical Group Colorectal Surgery Orlando Florida USA

**Keywords:** antibacterial, intra‐tumoral microbiota, nanomaterials, tumor therapy

## Abstract

Intra‐tumoral microbiota, which is a potential component of the tumor microenvironment (TME), has been emerging as a key participant and driving factor in cancer. Previously, due to technical issues and low biological content, little was known about the microbial community within tumors. With the development of high‐throughput sequencing technology and molecular biology techniques, it has been demonstrated that tumors harbor highly heterogeneous symbiotic microbial communities, which affect tumor progression mechanisms through various pathways, such as inducing DNA damage, activating carcinogenic pathways, and inducing an immunesuppressive environment. Faced with the harmful microbial communities in the TME, efforts have been made to develop new technologies specifically targeting the microbiome and tumor microecology. Given the success of nanotechnology in cancer diagnosis and treatment, the development of nanotechnology to regulate microscale and molecular‐scale interactions occurring in the microbiome and tumor microecology holds promise for providing new approaches for cancer therapy. This article reviews the latest progress in this field, including the microbial community within tumors and its pro‐cancer mechanisms, as well as the anti‐tumor strategies targeting intra‐tumoral microorganisms using nanotechnology. Additionally, this article delivers prospects for the potential clinical significance and challenges of anti‐tumor strategies against intra‐tumoral microorganisms.

## Graphical abstract

Intra‐tumoral microbiota, which is a potential component of the tumor microenvironment, has been emerging as a key participant and driving factor in cancer. This article reviews the latest progress in this field, including the microbial community within tumors and its pro‐cancer mechanisms, as well as the anti‐tumor strategies targeting intra‐tumoral microorganisms using nanotechnology. Additionally, this article provides prospects for the potential clinical significance and challenges of targeted anti‐tumor strategies against intra‐tumoral microorganisms.

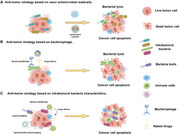

## 1 Introduction

Research on the tumor microenvironment (TME) represents a significant frontier in oncology. The TME refers to the specific ecological setting surrounding tumor cells, encompassing the tumor cells themselves, neighboring cells, extracellular matrix, microvasculature, intra‐tumoral microbiota, and the biomolecules interacting with these components [1]. While the intra‐tumoral microbiota was first observed and described in the 19th century, limitations in technology and understanding during the subsequent century have hindered a complete understanding of the breadth and depth of the tumor microbiome's impact [2]. Instead, the focus of research on the human microbiome then has predominantly been on the gut microbiota. Preclinical and some clinical evidence continuously emphasizes the role of the gut microbiota, especially in colorectal cancer patients, in response to chemotherapy and immunotherapy treatments [3]. In recent years, with the development of high‐throughput sequencing and other technologies, many studies have identified the microbial community composition in different types of tumors, such as colon cancer [4], lung cancer [5], breast cancer [6], and pancreatic cancer [7, 8] and so on. The composition and abundance of the microbial community in tumors are highly heterogeneous, and even the composition of the microbial community in tumors changes during tumor development [9].

The favorable conditions for microbial colonization in tumors include disrupted vascular systems and hypoxic yet nutrient‐rich immunosuppressive environments [10]. The interplay between intra‐tumoral microbiota, tumor immune microenvironment, and their interactions act as significant accomplices in tumor proliferation, metastasis, and drug resistance formation [11]. The functional roles of intra‐tumoral microbiota in tumor initiation and progression have garnered considerable attention in recent years.

Abundant research indicates that intra‐tumoral microbiota can promote cancer development through various pathways, such as inducing DNA damage, activating carcinogenic pathways, facilitating the formation of an immune‐suppressive microenvironment, and even impacting resistance to radiation, chemotherapy, and immunotherapy [12, 13]. In a groundbreaking review published by Douglas Hanahan in Cancer Discovery in 2022, titled “Hallmarks of Cancer: New Dimensions”, the importance of tumor microbiota in the TME was emphasized, with the microbial heterogeneity within tumors being listed as one of the key hallmarks of cancer [14]. The understanding of the complex relationships between different host‐intrinsic microorganisms and their multifaceted mechanisms affecting health and disease is considered scientifically and clinically significant, accelerating the development of novel therapeutic strategies targeting the microbiota to improve cancer treatment outcomes [15]. Previous microbiota modulation approaches, such as antibiotics, fecal microbiota transplantation [16], probiotics [17], dietary adjustments, and prebiotics, lacked specificity in targeting TMEs. For example, traditional antibiotic therapy, while often used adjunctively in cancer treatment, may lead to permanent bacterial elimination and dysbiosis, potentially with serious pathological consequences, when the balance between the bacteriostatic and bactericidal activities of antibiotics results in the eradication of bacterial species [18, 19]. Therefore, therapeutic strategies targeting the tumor microbiota need to meet the following criteria [20]: (1) the ability to regulate the complex TME, (2) specificity in regulating a particular tumor microorganism or the signaling pathways influenced by it, and (3) without affecting the balance of normal host microbiota. While the application of nanotechnology in microbiota modulation in cancer therapy is still in its infancy, some pioneering work highlights its tremendous potential. In this review, we summarize the latest findings on the composition of intra‐tumoral microbiota and its mechanisms in promoting cancer, and based on this, we explore the pioneering work of nanotechnology in improving cancer treatment through microbiota intervention.

## 2 Overview of the Cancer Microbiome

Microbiota refers to the collection of microorganisms and their genetic material that reside in a particular environment. The human body harbors various microorganisms including bacteria, fungi, viruses, and other microbes [21]. These microorganisms predominantly reside in areas such as the skin, oral cavity, nasal cavity, respiratory tract, digestive tract, and reproductive tract [22]. The microorganisms within the human body form complex symbiotic relationships with the host, playing crucial roles in maintaining human health, preventing diseases, and regulating immune responses. [23] In recent years, it has become increasingly clear that the ecosystem created by resident bacteria and fungi has profound effects on human health and disease, influencing many physiological processes, including digestion, metabolism, and even the development of cancer [24].

Research indicates that there are approximately 3.8 × 10^13^ microorganisms within the human body. Among them, about 97% of the microorganisms are intestinal bacteria, approximately 2%–3% reside outside the colon in areas such as the proximal gut, skin, lungs, and other tissues, while the remaining 0.1%–1% consist of archaea and fungi. The gene count of these microorganisms exceeds that of the human genome by 100 times, showcasing greater genetic diversity and flexibility [25].

Although most of the proposed links between cancer and microbiota have focused on the gut microbiota [26, 27], there is now ample evidence that the microbiota within tumors differs from that in healthy tissue. This progress is attributed to the development of high‐throughput sequencing technologies in recent years. The main technologies currently used for detecting intratumoral microorganisms include 16S rRNA sequencing and metagenomic sequencing. Both are based on sequencing microbial DNA. The 16S technology primarily focuses on 16S rDNA, while metagenomics covers all DNA. By combining these two technologies, one can leverage the cost‐effectiveness of 16S sequencing for the initial detection of the composition and diversity of a large number of samples. Target communities can then be selected for subsequent metagenomic research to uncover functional information about the communities. Based on this, the differences in the microbial communities between tumor and healthy regions have been disclosed, and it has been found microbiota may play a significant role in cancer diagnosis, pathogenesis, and treatment (Table 1).

**TABLE 1:** The composition and function of microbiota in different tumor types.

Tumor type	Microorganisms	Function
Colorectal cancer	*Fusobacterium* [64]	Related to tumor progression and metastasis
	*Escherichia coli* [51]	Causing DNA damage
	*Bacteroides, Roseburia, Ruminococcus, Oscillibacter, Porphyromonas, Peptostreptococcus, Parvimonas* [33]	Related to tumor progression
	*Parvimonas, Prevotella* [31]	
Gastric cancer	*Peptostreptococcus stomatis*, *Dialister pneumosint*es, *Slackia exigua*, *Parvimonas micra*, and *Streptococcus anginosus* [40]	Related to tumor progression
	Proteobacteria, Firmicutes, Bacteroidetes, Fusobacteria*, and* Actinobacteria	
	*Methylobacterium* [41]	Gastric carcinogenesis.
	*Streptococcus anginosus, Streptococcus constellatus* [42]	Early diagnosis and screening
Lung cancer	*Streptococcus, Veillonella* [43]	Related to upregulation of PI3K.
	*Acidovorax* [5]	Related to TP53 mutation
	*Thermus* [107]	Related to tumor progression
Melanoma	*Acinetobacter, Actinomyces, Corynebacterium, Enterobacter, Streptococcus* [44]	
Pancreatic cancer	*Pseudomonas aeruginosa, Streptomyces, yeast spores, Clostridium perfringens* [49]	Diagnosis and screening
	*Escherichia coli, Klebsiella pneumoniae, Citrobacter freundii, Clostridium perfringens, and Enterobacter cloacae* [108]	Related to tumor progression
	*Gammaproteobacteria* [7]	Inducing chemotherapy resistance
Breast cancer	Actinobacteria, Firmicutes, Proteobacteria [109]	
	*Pseudomonas, Azomonas, Porphyromonas* [47]	
	*Fusobacterium* [110]	Related to tumor progression
Cervical cancer	*Lactobacillus iners* [48]	Inducing chemotherapy and radiation resistance

The colon is one of the richest organs in terms of microbial abundance in the human body, containing an estimated 70% of the human microbiome [28]. The microbial community within the colon plays crucial roles in aiding digestion and nutrient absorption, maintaining the integrity of the intestinal mucosal barrier, and protecting the host from invasion by pathogenic microorganisms [29]. Abnormalities in the colonic microbial community may lead to the development of intestinal diseases and may also play significant roles in the progression of cancer [30]. By using 16S rRNA sequencing technology, samples from 59 patients undergoing CRC surgery, 21 patients with polyps, and 56 healthy controls were analyzed. The results revealed significant differences in the mucosal microbiota composition between cancer patients and controls. Specifically, cancer patients showed an increased abundance of operational taxonomic units (OTUs), mainly including *Bacteroides*, *Ruminococcus*, *Oscillibacter*, and *Roseburia*, and some genera previously reported as oral pathogens, such as *Fusobacterium* [28, 31]. In colorectal cancer, the levels of tumor‐associated bacteria like *Clostridium*, *Bacteroides*, *Parvimonas*, and *Prevotella* have been observed to fluctuate across different stages of colon and rectal cancer progression. This suggested that alterations in the microbiota played a significant role in the advancement of colon and rectal cancer [32, 33]. Research has shown that *Fusobacterium*, which is currently hailed as a star bacterium in colorectal cancer research, contributes to the formation and advancement of both colon adenomas and colon cancer, [34, 35] and it has also been detected in nodal and distant metastasis in patient samples [36, 37].

The stomach, as an organ of the digestive tract, harbors a rich microbial community, with *Helicobacter pylori* playing a well‐documented role in the development of gastric cancer [38]. Moreover, research has revealed that Proteobacteria, Firmicutes, Bacteroidetes, Fusobacteria, and Actinobacteria were the most abundant groups in gastrointestinal cancer, with a bacterial composition significantly distinct from that in normal tissue [39]. Through 16S rRNA gene analysis of 81 gastric mucosal samples, including superficial gastritis (SG), atrophic gastritis (AG), intestinal metaplasia (IM), and gastric cancer (GC), it was found that the mucosal microbiota was significantly dysregulated in IM and GC subjects. Bacteria such as *Peptostreptococcus stomatis*, *Dialister pneumosintes*, *Slackia exigua*, *Parvimonas micra*, and *Streptococcus anginosus* might play important roles in the progression of GC [40]. Rui Peng et al. found through sequencing analysis of patient fecal and tissue samples that *Firmicutes* were the dominant phylum in the feces of gastric cancer and chronic gastritis patients, while Proteobacteria were the most dominant phylum in the tissues of gastric cancer and chronic gastritis patients. Additionally, *Methylobacterium* was associated with poor prognosis in gastric cancer [41]. The reaserch has shown that *Streptococcus anginosus* (*Sa*) and *Streptococcus constellatus* (*Sc*) were significantly enriched in the mucosa and feces of patients with precancerous lesions and early‐stage gastric cancer. Independent validation in a separate cohort confirmed the superior accuracy and sensitivity of characteristic fecal *Sa* and *Sc* in diagnosing early and advanced GC [42].

The lung, being a mucosal organ, is exposed to a diverse microbial community. Numerous studies have employed metagenomic sequencing technology to categorize bacteria associated with lung cancer. Research has shown that analysis of lower respiratory tract and oral samples reveals an abundance of *Streptococcus* and *Veillonella* in lung cancer airway samples compared to healthy controls [43]. Interestingly, Greathouse et al. detected the lung tissue microbiota in 33 healthy individuals and 142 lung cancer patients and found a unique lung microbiota in lung cancer patients. Among them, *Acidovorax* is more abundant in smokers [5].

The skin is another organ with an abundant microbiome, which leads to the exposure of skin cancer to numerous microorganisms. In normal skin tissue, *Corynebacteria*, *Propionibacteria*, and *Staphylococci* are the three main genera of commensal microbiota, while the composition of microbiota is strongly connected with its location. An investigation identified bacteria belonging to the *Enterobacter*, *Acinetobacter*, *Actinomyces*, *Corynebacterium*, and *Streptococcus genera* in melanoma samples [44]. Additionally, peptides originating from bacteria have been detected in melanoma, offering promising prospects as targets for anti‐tumor therapy [45].

There is a unique microbiome present in breast cancer tissue [46], with certain cancers and even breast cancer subtypes exhibiting characteristic changes, while breast cancer tissue has more *Pseudomonas*, *Azomonas*, and *Porphyromonas* [47]. Cervical cancer, as a female cancer, is closely associated with the disruption of vaginal microbiota balance. Research has found that a species of *Lactobacillus*, *L. iners*, which produces L‐lactic acid, was associated with reduced survival rates in cervical cancer patients. It induced chemotherapy and radiotherapy resistance in cervical cancer cells and leads to metabolic rewiring or alterations in multiple metabolic pathways within tumors [48].

Pancreatic cancer, being one of the most malignant types of cancer, has garnered significant attention regarding its tumor microbiome composition. Erick Riquelme et al. analyzed the tumor microbiome composition in short‐term and long‐term survival patients with PDAC and found higher α‐diversity in the tumor microbiome of LTS patients and identified specific tumor microbiome features including *Pseudomonas aeruginosa*, *Streptomyces*, *yeast spores*, and *Clostridium perfringens*, which were highly predictive of long‐term survival in both discovery and validation cohorts [49]. Geller et al. found common bacteria in pancreatic cancer, including *Escherichia coli*, *Klebsiella pneumoniae*, *Citrobacter freundii*, *Clostridium perfringens*, and *Enterobacter cloacae*, through 16s rRNA sequencing [8].

Detection of the tumor microbiome relies on the development of high‐throughput sequencing technologies and analytical methods. Due to the low biomass of intra‐tumoral microbiota, environmental contamination during sampling, and strong host background, studies on the microbiome composition of the same cancer type may yield different results. This necessitates continuous improvement in sampling procedures and decontamination algorithms by researchers. Currently, research on the precise detection of the tumor microbiome in more rare cancer types and different cancer subtypes is underway, and it is believed that in the future, tumor microbiome profiles relevant to cancer will emerge.

## 3 Mechanisms of Intratumor Microbiota Affecting Tumorigenesis and Treatment

Microorganisms can inhabit tissues and tumors within an organism because of the favorable environment created by neoplastic/cancerous and precancerous lesions. These lesions provide optimal conditions for microorganism colonization and long‐term survival. The accelerated angiogenesis and tumor necrosis linked to these lesions foster the formation of a highly hypoxic and nutrient‐rich TME, promoting the selective colonization of facultative and/or anaerobic bacterial strains [50]. These microorganisms have significant effects on cancer development, progression, therapy response, and antitumor immunity through various mechanisms.

The targets of tumor microbiota, their secreted factors, and metabolic products mainly include epithelial cells, tumor cells, and immune cells. It has been suggested that specific microbiota might directly cause DNA damage in epithelial cells, thus contributing to tumor initiation and progression. Moreover, certain microbiota can elicit pro‐inflammatory responses and activate carcinogenic pathways. Tumor‐associated microbiota may also promote tumor progression by inducing immune suppression, thereby influencing cancer therapy in various ways. Some microbiota own the ability to metabolize anti‐cancer drugs, while changes in antitumor immunity could impact the effectiveness of cancer treatment. Therefore, exploring the role of tumor‐associated microbiota is crucial for understanding the mechanisms underlying tumor development and progression, developing innovative therapeutic approaches, and improving treatment outcomes.

### 3.1 Induction of DNA Damage

**FIGURE 1:**
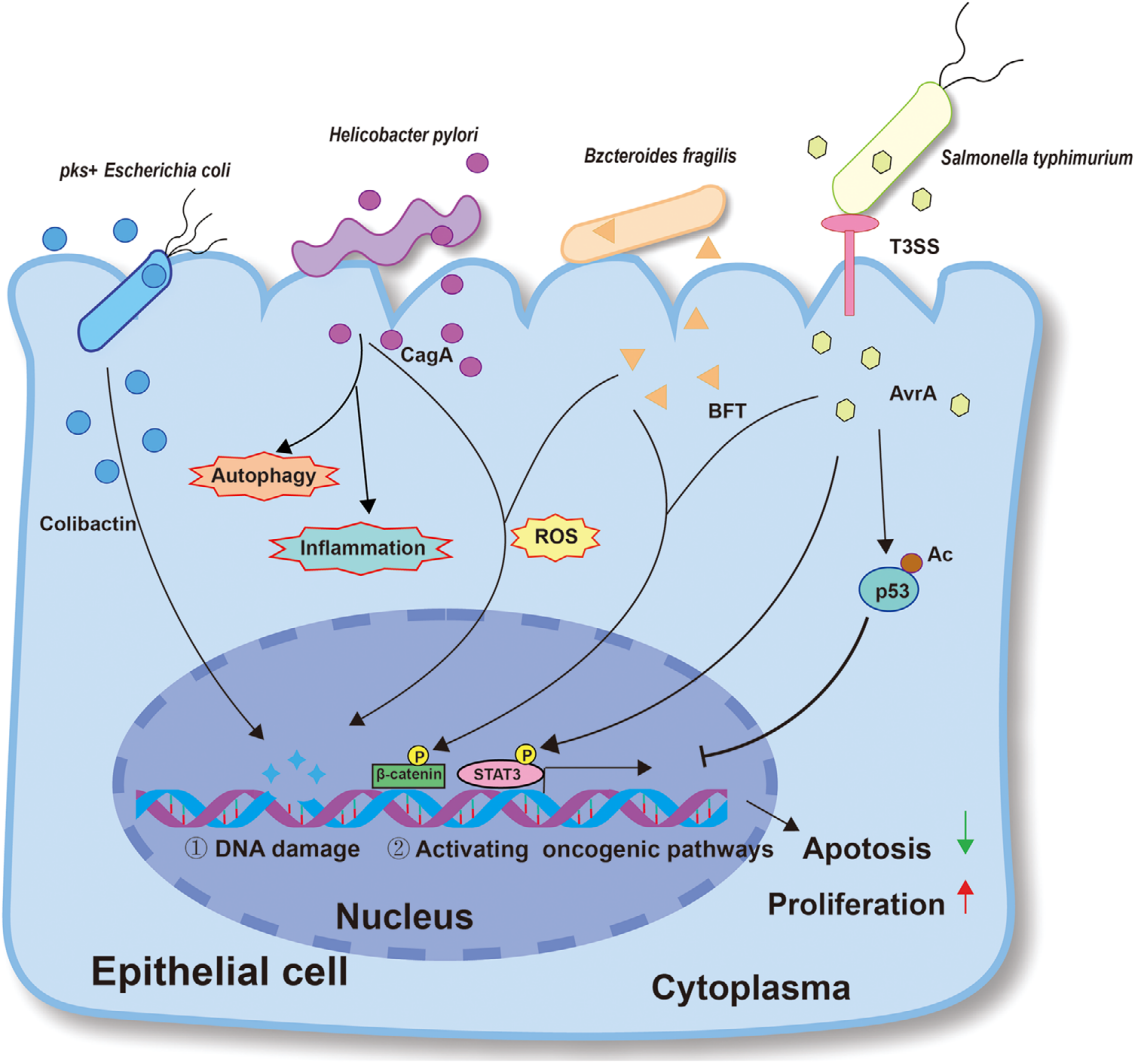
Mechanisms of intratumor microbiota affecting tumorigenesis and treatment in an epithelial cell.

Numerous microorganisms have developed the ability to generate substances that have the potential to induce DNA damage, halt the cell cycle, and provoke genetic instability. For instance, specific strains of *E. coli* and other members of the *Enterobacteriaceae* family containing *pks* pathogenicity islands can generate colibactin, a compound capable of inducing double‐stranded DNA damage to facilitate the progression of colorectal cancer [51]. In addition to directly inducing DNA damage, tissue‐resident microorganisms such as EspF‐expressing *E. coli* [52] and *H. pylori* [53, 54] can disrupt mechanisms of DNA mismatch repair to further exacerbate genomic instability and drive tumorigenesis. *Bacteroides fragilis* is commonly found colonizing the colon, but it has also been found in breast cancer tissues. Its virulence is associated with a 20‐kDa zinc metalloprotease toxin known as the *B. fragilis* toxin, which activates the β‐catenin signaling pathway [55]. *Fusobacterium nucleatum* can release a toxin that initiates double‐stranded DNA breaks to induce DNA damage [56]. T3SS stands for Type III Secretion System, which is a tool utilized by Gram‐negative bacterial pathogens to inject virulence proteins directly into infected host cells [57]. *Salmonella Typhimurium* can inject AvrA through T3SS, thereby mediating the acetylation of p53 [58] and the phosphorylation of STAT3 [59], promoting the activation of downstream oncogenes. These events, which can occur during the progression of epithelial cells to cancer cells, are likely to be the contributing factors to carcinogenesis (Figure 1).

### 3.2 Activation of Oncogenic Pathways

**FIGURE 2:**
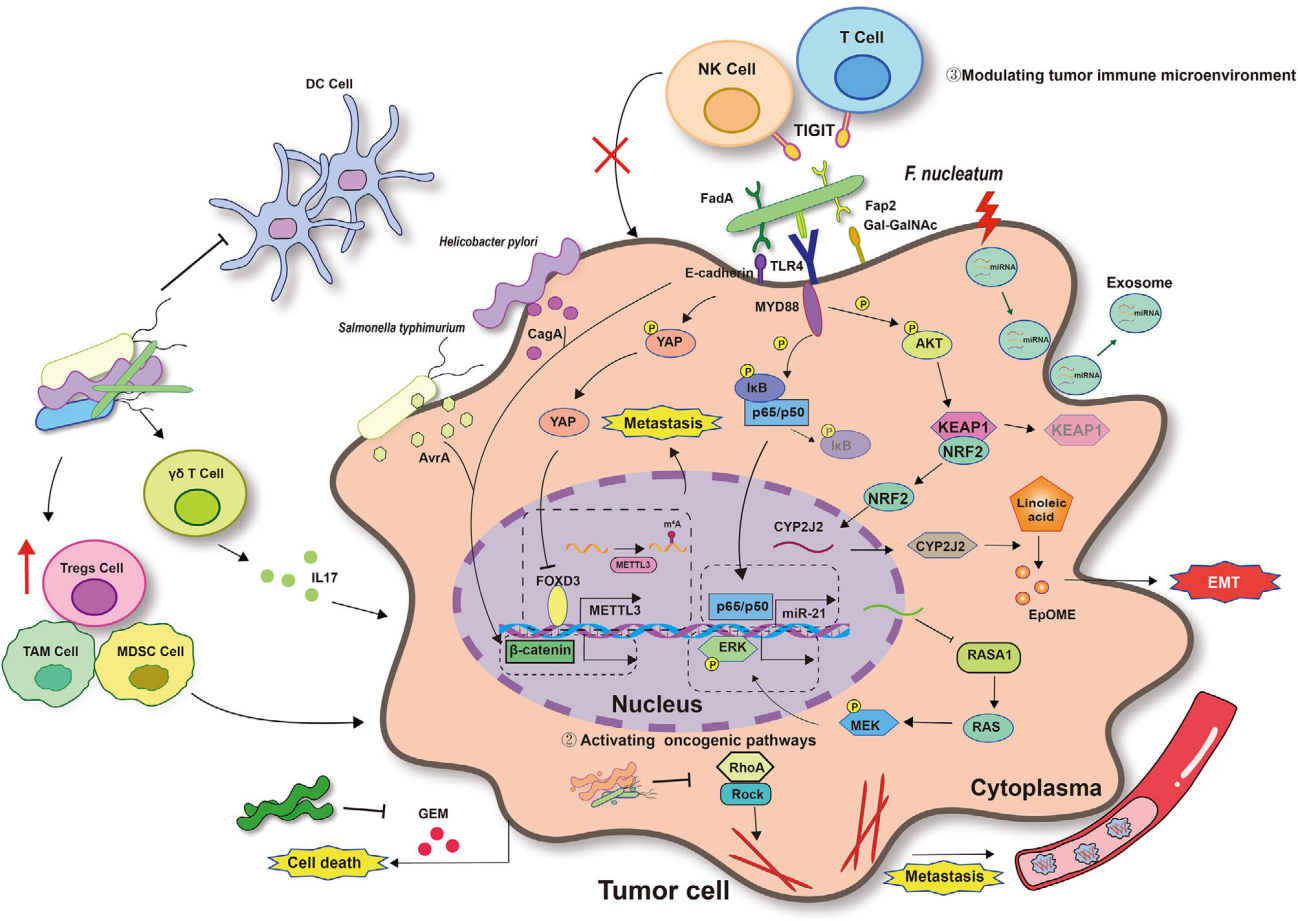
Mechanisms of intratumor microbiota affecting tumorigenesis and treatment in a tumor cell.

The role of tumor microbiota in epithelial cells is also present in tumor cells (Figure 2). Several studies suggest that microbiota can activate NF‐𝜅B or β‐catenin pathways, promoting tumor progression [60]. Moreover, certain Toll‐like receptor members, such as TLR4, play a significant role in the crosstalk between tumors and microbiota [61, 62]. For instance, research indicates that *F. nucleatum* targets TLR4 and MYD88 innate immune signaling pathways, as well as specific microRNAs to activate the autophagy pathway and modify the response to chemotherapy in colorectal cancer [62]. Kong et al. identified an additional oncogenic pathway in *F. nucleatum*, which promoted colorectal cancer development by elevating the levels of CYP2J2 and 12,13‐EpOME through the activation of the TLR4/Keap1/NRF2 signaling pathway. This pathway contributes to the invasion and metastasis of colorectal cancer [63]. Furthermore, the tumor microbiota also influences tumor epigenetics. For instance, *Fusobacterium* induces a notable reduction in m^6^A modification in colorectal cancer cells and the PDX model by suppressing the expression of m^6^A methyltransferase METTL3, thereby promoting the invasion and metastasis of colorectal cancer [64]. In addition, Fn infection may stimulate tumor cells to produce exosomes rich in miR‐1246/92b‐3p/27a‐3p, which are transported to uninfected cells to promote tumor cell proliferation and metastasis [65].

### 3.3 Modulating the Tumor Immune Microenvironment

Microbes, as significant components of the TME, also play a regulatory role in immune cells within the microenvironment. Immune checkpoint blockade (ICB), as a current hot spot strategy for anti‐tumor treatment, has attracted widespread attention (Figure 2). Research has demonstrated that Fap2 expressed by *F. nucleatum* could directly interact with the immune checkpoint protein TIGIT, thereby suppressing the anti‐tumor activity of NK cells and T cells [66]. Additionally, studies have revealed that bacterial eradication could increase PD‐1 expression, improve the effectiveness of immunotherapy, and reshape the immunological landscape of the pancreatic cancer TME, which included diminishing myeloid‐derived suppressor cells, enhancing M1 macrophage differentiation, and fostering the activation of CD4^+^ T cells and CD8^+^ T cells [67].

Microorganisms may achieve this through conditional activation of pattern‐recognition receptors. For example, bacterial lipopolysaccharide, which was found within both cancer cells and immune cells in the TME [68], can bind to TLR4 not only on infiltrating monocytes to skew their differentiation to an immunosuppressive M2 phenotype [69, 70] but also on tumor cells to promote recruitment of CD11bGr1 myeloid‐derived suppressor cells and CD11dCD5 regulatory B cells, collectively suppressing local antitumor T cell responses [71]. Moreover, Jin et al. have identified that alterations in lung microbiota trigger the production of Myd88‐dependent IL‐1β and IL‐23 by myeloid cells. These cytokines stimulated the activation and proliferation of Vy6^+^Vδ1^+^γδ T cells, resulting in the secretion of IL‐17 and IL‐22, which promote inflammation and neutrophil infiltration [72].

Moreover, bacteria play important roles in resistance to chemotherapy and radiotherapy. For instance, in pancreatic ductal adenocarcinoma, *Leore demostreated Gammaproteobacteria* could metabolize the chemotherapeutic drug gemcitabine into its inactive form, depending on bacterial enzyme cytidine deaminase expression [7]. On the other hand, radiotherapy of tumor lesions was more effective when vancomycin eliminated Clostridiales‐derived immunosuppressive metabolites (butyrate and propionate) putatively by increasing DC antigen presentation and concomitant CD8^+^T cell priming [73].

Furthermore, Fu et al. found that tumor‐infiltrating bacteria carried by circulating tumor cells in breast cancer enhanced resistance to fluid shear stress by inhibiting the activation of RhoA and ROCK, thereby reorganizing the actin cytoskeleton of host cells to promote their survival and facilitate distant metastasis of tumor cells [74]. While many reports have suggested the significant role and intricate mechanisms of tumor microbiota in cancer initiation and progression, the vast majority remained unknown, necessitating further research for elucidation.

## 4 Nanotechnology‐Based Strategies Targeting Intratumoral Microorganisms for Cancer Therapy

**FIGURE 3:**
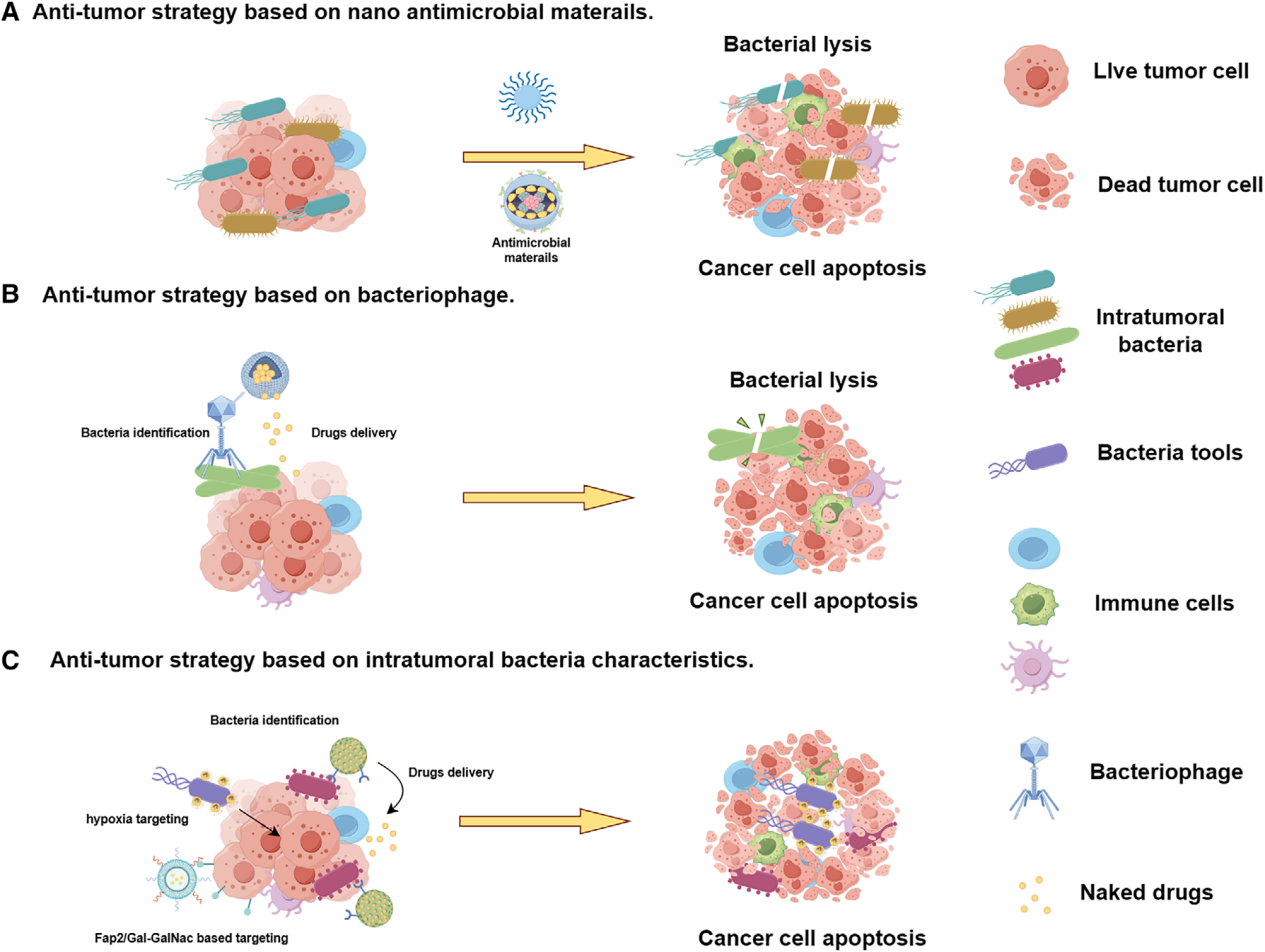
Nanotechnology‐based strategies targeting intratumor microbiota for cancer therapy.

The continuous advancement of nanotechnology has facilitated the development of drug delivery systems, enhancing drug accumulation and reducing side effects. Nano‐therapeutic approaches have been applied to target intra‐tumoral microorganisms, using nanocarriers as effective antimicrobial drug delivery systems. Currently, there are three main strategies for using nanotechnology to eliminate intertumoral microorganisms in anti‐tumor research (Figure 3; Table 2):

**TABLE 2:** Nanotechnology‐based strategies targeting intratumoral microorganisms for cancer therapy.

Antimicrobial materials	Antibacterial compound	Bacteria	Cancer type	Combination Therapy
Nanoparticles of chitosan‐glutamic acid conjugates [81]	Antibiotics	*Helicobacter pylori*	Gastric cancer	
Amoxicillin nanoparticles [79]	Antibiotics	*Helicobacter pylori*	Gastric cancer	
MTI‐FDU [84]	Antibiotics	*Fusobacterium nucleatum*	Colorectal cancer	Chemotherapy
sNP@G/IR [85]	Antibacterial nanomaterials	Intracellular bacteria	Pancreatic cancer	Chemotherapy
Nb2C/Au/Anti‐TNFα [87]	Metal ions	Intracellular bacteria	Colorectal and Breast cancer	Inhibitors
Au@BSA‐CuPpIX [88]	Metal ions	*Fusobacterium nucleatum*	Colorectal cancer	SDT
OLP/PP [56]	Saturated fatty acid	*Fusobacterium nucleatum*	Colorectal cancer	Chemotherapy
D‐IDNPs‐A [90]	Bacteriophages	*Fusobacterium nucleatum*	Colorectal cancer	Chemotherapy
M13@Ag [91]	Bacteriophages/ Metal ions	*Fusobacterium nucleatum*	Colorectal cancer	ICB/Chemotherapy
YB1‐INPs [94]	Thermal energy	*Salmonella typhimurium*	Bladder cancer	PTT
AGS‐NPs [95]	Antibiotics	*Helicobacter pylori*	Gastric cancer	
LTA‐MSNs [98]		Intracellular bacteria	Colon, Lung, and Breast cancer	Chemotherapy
Colistin‐LipoFM [101]	Antibiotics	*Fusobacterium nucleatum*	Colorectal cancer	ICB

### 4.1 Anti‐Tumor Strategy Based on Antimicrobial Materials

The current situation of bacterial drug resistance caused by antibiotics is a global public health crisis. The overuse and misuse of antibiotics have led to the emergence of antibiotic‐resistant bacteria, which can cause severe infections that are difficult or impossible to treat [75]. According to the World Health Organization (WHO), antibiotic resistance is one of the biggest threats to global health today [76]. Moreover, antibiotics can also cause some side effects, including diarrhea, liver damage, neurotoxicity, ototoxicity, nephrotoxicity, and blood disorders, which may vary depending on the type of drug, duration of use, and the patient's health status [77, 78]. Therefore, developing new and multidimensional strategies to combat microbial infections is warranted. These include: (i) modification of existing antibiotics, (ii) searching for new and novel antibiotics, (iii) development and improvement of antibiotics carrier system to reduce the amount and frequency of antibiotic doses, and (iv) the development of targeted antibiotic delivery systems. Nanotechnologies have been used to load and deliver antibiotics to kill bacteria, especially cancer‐causing bacteria [79, 80, 81]. After being nanosized, antibiotics can accumulate at tumor sites, avoiding widespread distribution throughout the body. By using lower doses, they can effectively clear intra‐tumor bacteria while not disturbing the normal balance of gut microbiota and causing serious side effects. Ramteke et al. developed and characterized targeted sustained release nanoparticles of chitosan‐glutamic acid conjugates containing triple therapy (amoxicillin, clarithromycin, and omeprazole) for *H. pylori* to improve its therapeutic effect and reduce its dose‐related side effect [80]. Chang et al. incorporated amoxicillin into pH‐sensitive hydrogel nanoparticles to eradicate *H. pylori* infection in the stomach [79].

**FIGURE 4:**
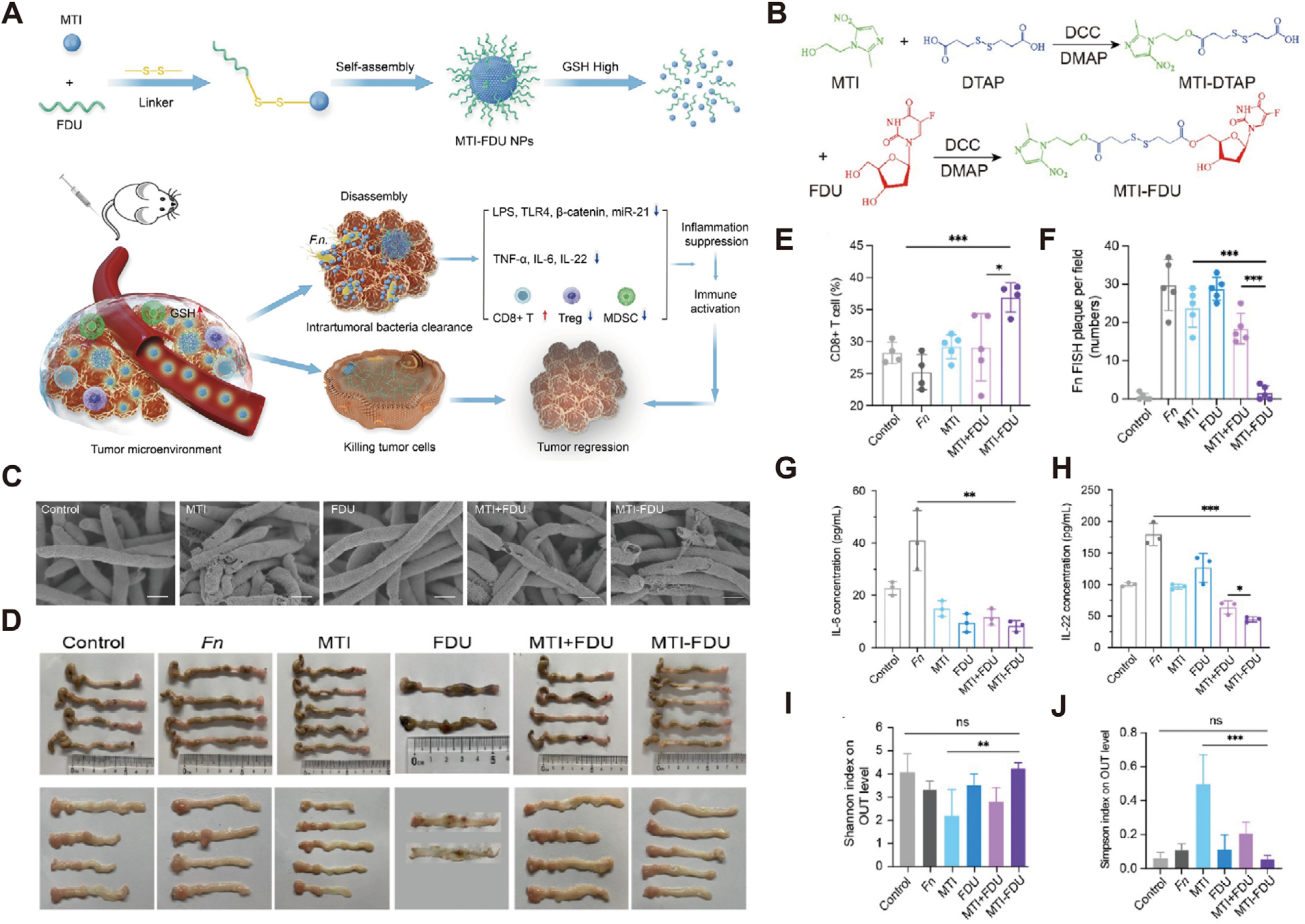
Design and therapeutic outcomes of MTI‐FDU for *Fusobacterium nucleatum*‐induced drug‐resistant CRC. (A) Schematic illustration of MTI‐FDU for tumor targeting delivery. (B) Synthetic routes of MTI‐FDU. (C) SEM micrographs of Fn treated with PBS (control), MTI, FDU, MTI+FDU, and MTI‐FDU for 12 h. Scale bar = 1 µm. (D) General view of mouse colorectum. (E) Quantitative analysis of the percentage of CD8+ T cells in different groups. (F)Quantitative analysis of Fn FISH plaques in different groups. (G,H) Cytokine levels of IL‐6 and IL‐22 in intestinal tissue were detected through ELISA among different treated groups (*n* = 3). (I,J) Analysis of alpha diversity coefffcient of intestinal flora, Shannon index (I) and Simpson index (J) were observed by 16S rDNA sequencing (*n* = 3) [84]. Copyright 2023 American Chemical Society.

Antibiotic‐based nano‐delivery systems also involve the nanosized antibiotics and their combination with chemotherapeutic drugs or photothermal agents, synergistically combating bacterial infections and tumors [82, 83]. A dual‐therapy nanoparticle comprising a combination of metronidazole and fluorouridine was constructed. These nanoparticles could be released under high glutathione levels in the TME, leading to the disruption of pro‐tumor bacteria *F. nucleatum* enriched in colorectal cancer and the killing of tumor cells (Figure 4A). Metronidazole was conjugated with fluorouridine to form the amphiphilic small molecule, which further self‐assembled in aqueous solution to form metronidazole–fluorouridine nanoparticles (MTI‐FDU) (Figure 4B). SEM assay found MTI‐FDU had an antibacterial effect equal to that of MTI bare drug (Figure 4C). In vivo, nanodrug MTI‐FDU displayed apparent suppression of tumor cells (Figure 4D) which reshaped the immune microenvironment through clearance of bacteria (Figure 4E) in the CRC spontaneous AD/Fn model, including promoting the infiltration of CD8^+^ T cells (Figure 4F) and reducing the secretion of inflammatory cytokines IL‐6 (Figure 4G) and IL‐22 (Figure 4H). Compared to the free metronidazole, it did not disturb the balance of intestinal microbiota (Figure 4I,J) [84].

**FIGURE 5:**
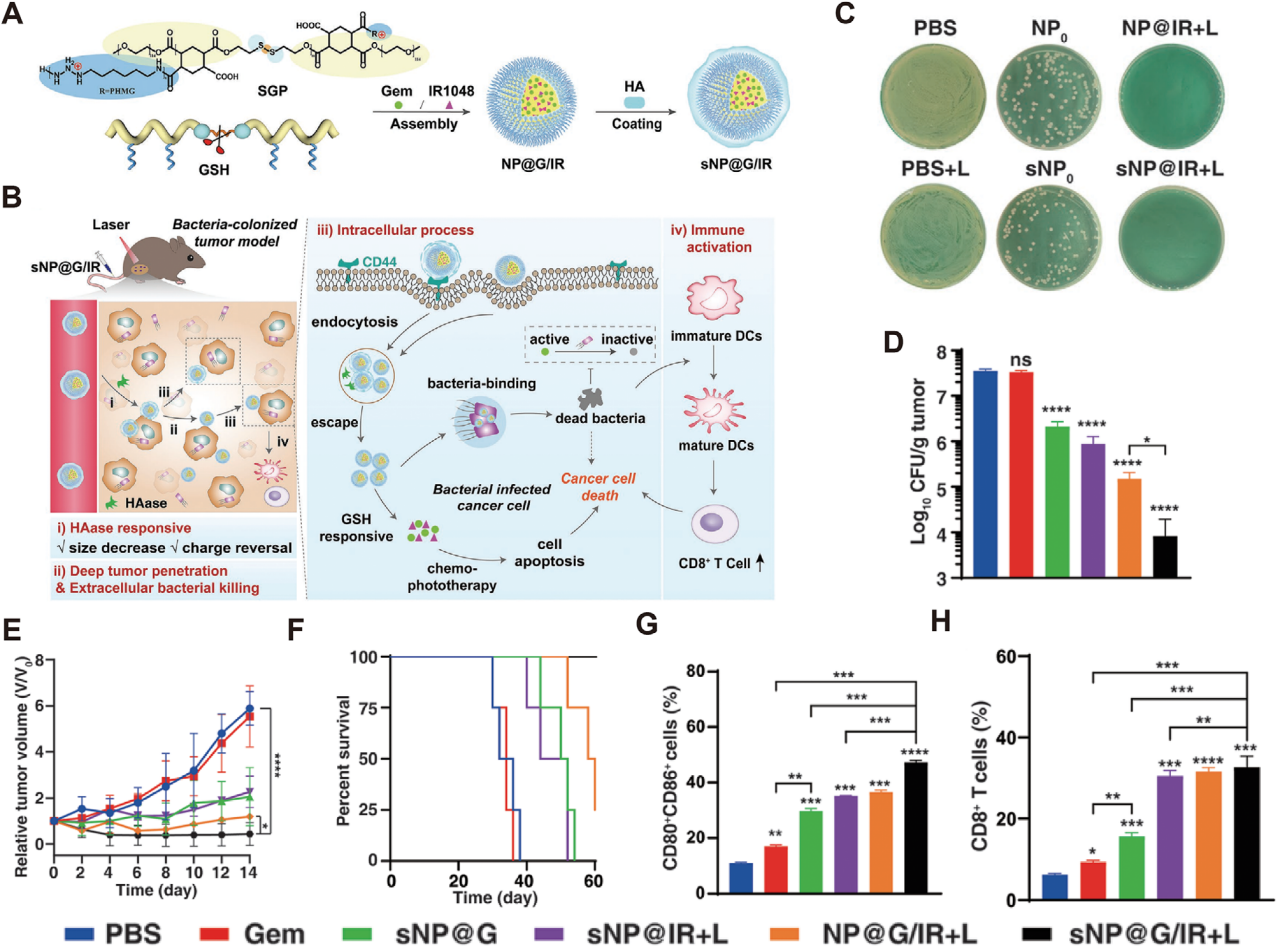
Strategy and therapeutic outcomes of dual‐cascade responsive nanoparticles for bacteria‐colonized tumor. (A) Preparation process of the dual‐cascade responsive sNP@G/IR with core–shell structure. (B) sNP@G/IR suppresses bacteria‐colonized tumor growth by eliminating tumor‐resident intracellular bacteria and precise drug delivery. (C) Colony plate images of EcN treated with various drugs at 8 h in LB medium (*n* = 3). (D) CFU in tumors of mice receiving different treatments (*n* = 3). (E) Relative tumor volume curves of EcN‐colonized tumors in mice after different treatments (*n* = 4). (F) Survival fractions of mice after different treatments within 60 days. (G) Mature DCs in tumors. (H) CD8+ T cells in tumors [85]. Copyright 2022 Wiley‐VCH GmbH.

In addition to antibiotics, there are currently various antibacterial nanomaterials, including polymers and inorganic metals (Figure 3A). For example, Xiaoxu Kang et al. developed a dual‐responsive nanoparticle, sNP@G/IR, composed of a hyaluronic acid (HA) shell and a glutathione (GSH)‐responsive polymer core that encapsulates gemcitabine (Gem) and a photothermal agent (IR1048) (Figure 5A). The nanoparticle targets CD44‐expressing tumor cells via HA and, upon degradation by hyaluronidase in the extracellular matrix, undergoes size reduction and charged reversal to penetrate deep into the tumor (Figure 5B). The exposed guanidine groups on NP@G/IR could kill intracellular bacteria (Figure 5C,D) In vivo and in vitro by disrupting the cell membrane. In vivo, the Gem was protected and subsequently released in a responsive manner. Under laser irradiation, the photothermal therapy of IR1048 further eliminated the tumor and bacteria, which delayed tumor progression (Figure 5E), improved the survival rate (Figure 5F), and activated immune responses (Figure 5G,H), achieving the designed “attack–defense maneuver” and combination therapy [85].

**FIGURE 6:**
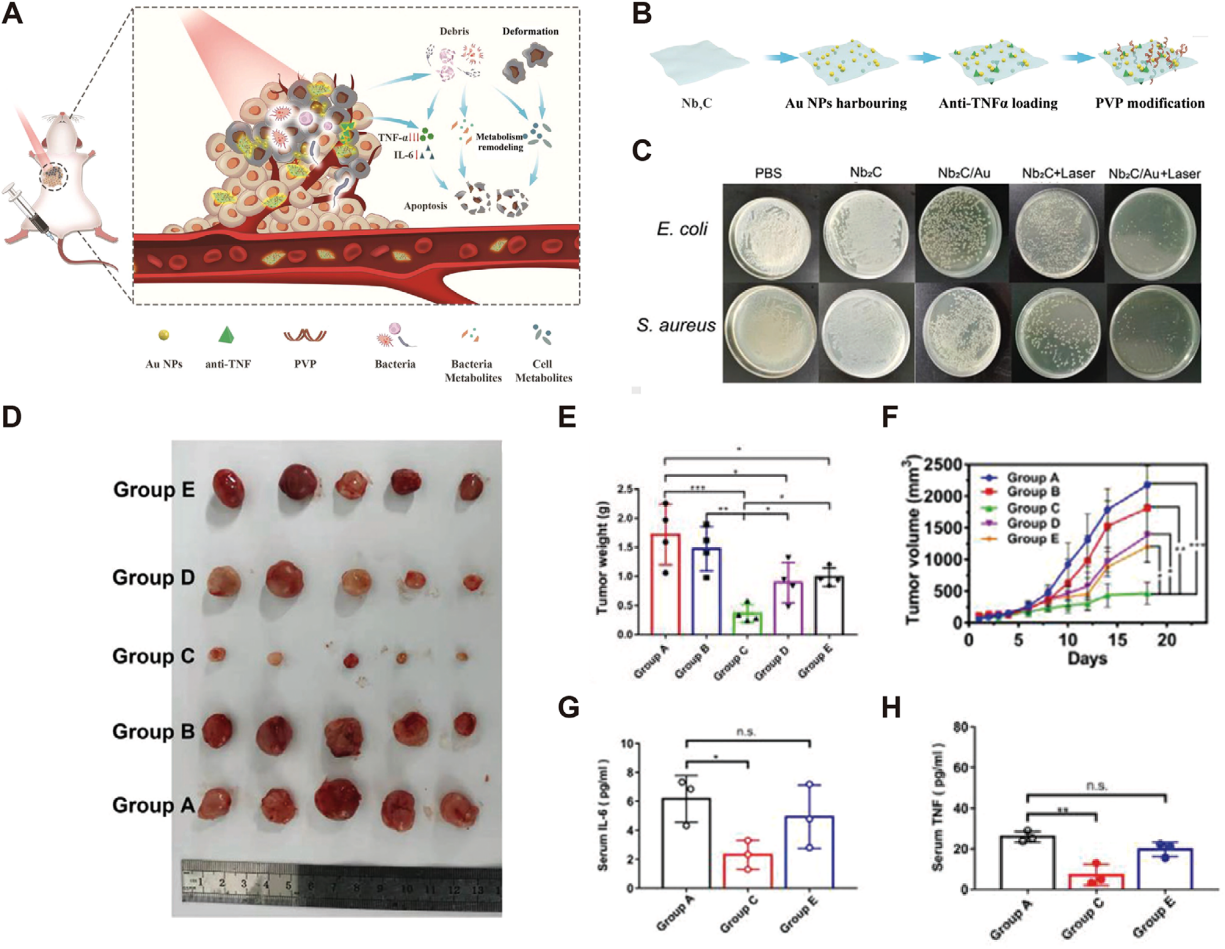
A microbiome metabolism–engineered phototherapy strategy designed to achieve both “chemical” and “physical” bacterial regulations via altering the abundance, diversity of intratumoral microbiome, and disrupting inflammation and metabolic pathways of microbiome and tumor microenvironment. (A) The detailed design principle of antibacterial and anti‐inflammatory actions using such Nb2C/Au/anti‐TNFα‐PVP nanocomposite to reshape tumor metabolic microenvironment. (B) Schematic for depicting synthesis procedures of Nb2C/Au‐anti‐TNFα‐PVP nanocomposite. (C) Digital photos of *Escherichia coli* (top panel) and *Staphylococcus aureus* (bottom panel) bacteria in agar plates where bacteria were re‐cultivated after different treatments (PBS, Nb2CPVP, Nb2C/Au‐PVP, Nb2C‐PVP+Laser, and Nb2C/Au‐PVP+Laser) for 4 h. Digital photos (D) and weights (E) of harvested tumors from CT26 tumor‐bearing mice that experienced corresponding treatments in Groups A–E at the end of the experimental period. *Note*: Groups A–E represent Control, Laser alone, Nb2C/Au/anti‐TNFα‐PVP+Laser, Nb2C/Au/anti‐TNFα‐PVP+Laser (cage change), and Nb2C/Au/‐PVP+Laser, respectively, and in Group D, the treatment method was identical to that of Group C, but after treatment, mice were transferred to the fecal environment of tumor‐bearing mice in Group A (*n*=4). (F) Time‐dependent tumor growth profiles of CT26 tumor‐bearing mice model after having experienced corresponding treatments in Groups A–E. (G,H) The expression levels of serum IL‐6, TNF in Group A, Group C, and Group E using ELISA assay method (*n*=3) [87]. Copyright 2022 Wiley‐VCH GmbH.

Metal ions are known to achieve bactericidal effects by disrupting bacterial cell membranes, precipitating proteins, ion exchange, antioxidative stress, and oxidation [86]. Polymer‐based drugs and inorganic metal nanodrugs mainly use certain antibacterial materials as nanodrug carriers, forming particles with nanoscale size by encapsulating, dispersing, non‐covalently or covalently binding chemotherapy drugs, etc. Fanlei Kong et al. developed a composite nanomedicine, Nb2C/Au/Anti‐TNFα, by combining a super‐thin Nb2C photothermal agent with antibacterial gold nanoparticles and anti‐TNF‐α drugs via electrostatic adsorption (Figure 6A,B). Nb2C/Au‐PVP+Laser group showed the best antibacterial effect in vitro (Figure 6C). In vivo, the nanomedicine eliminated intratumoral bacteria and killed tumor cells with a combination of the anti‐TNF‐α drug under the laser (Figure 6D–F). Additionally, the inflammatory cytokine levels (IL‐6, TNF‐α) in mice serum were tremendously down‐regulated (Figure 6G,H) [87].

Moreover, an antibacterial nanoplatform Au@BSA‐CuPpIX was constructed, which generates reactive oxygen species (ROS) under ultrasound and effectively eliminates *F. nucleatum*. It enhances the efficacy of sonodynamic therapy (SDT) for orthotopic colorectal cancer (CRC) and inhibits lung metastasis. Moreover, it reduces the phototoxicity of metalloporphyrin accumulated in the skin during tumor treatment, preventing severe inflammation and skin damage, thereby promoting the clinical translational potential of SDT [88].

Lauric acid is a type of saturated fatty acid with a 12‐carbon chain and has antimicrobial properties [89]. Xiaohui Li et al. developed a novel tumor‐targeting pH‐responsive nanomaterial for delivering the antibacterial fatty acid lauric acid (LA) and the chemotherapeutic drug oxaliplatin (OXA) to effectively eliminate extracellular and intracellular *Fn* in the acidic TME. The release of OXA significantly inhibited tumor growth [56].

### 4.2 Anti‐Tumor Strategy Based on Bacteriophage Targeting of Bacteria Within Tumors

Bacteriophages are viruses that infect bacteria. They are composed of genetic material, either DNA or RNA, enclosed in a protein coat. Phages are highly specific in their host range, meaning that they can only infect certain types of bacteria. Phages have shown great potential as an alternative to antibiotics in the treatment of bacterial infections, particularly those caused by antibiotic‐resistant bacteria. Phage therapy involves using specific phages to target and eliminate pathogenic bacteria while leaving beneficial bacteria unharmed. Currently, there are articles that combine bacteriophages and nanocarrier systems to specifically target bacteria‐enriched microbiota in cancer. By actively targeting bacteria through bacteriophage binding, the loaded nanodrugs are expected to be delivered to the tumor site, exerting an anti‐tumor effect (Figure 3B).

Diwei Zheng et al. isolated a bacteriophage strain from human saliva that could specifically lyse fusobacterium. They then encapsulated the first‐line colon cancer drug, irinotecan (IRT), in dextran nanoparticles (DNPs) to form IRT‐loaded DNPs (IDNPs). Subsequently, they used a bioorthogonal reaction to covalently link DNP‐modified with dibenzocyclooctyne (DBCO) to the phage modified with azide to construct a bacteriophage‐guided bio‐inorganic hybrid nanocarrier system (D‐IDNPs‐A). The phage in this drug delivery system could target colon tumors enriched with *Fn* and clear *Fn*, thereby overcoming chemoresistance to IRT in colon cancer tumors mediated by *Fn* and improving the chemotherapy efficacy. In addition, DNPs can promote the proliferation of endogenous butyrate‐producing bacteria, reducing the side effects of chemotherapy [90].

**FIGURE 7:**
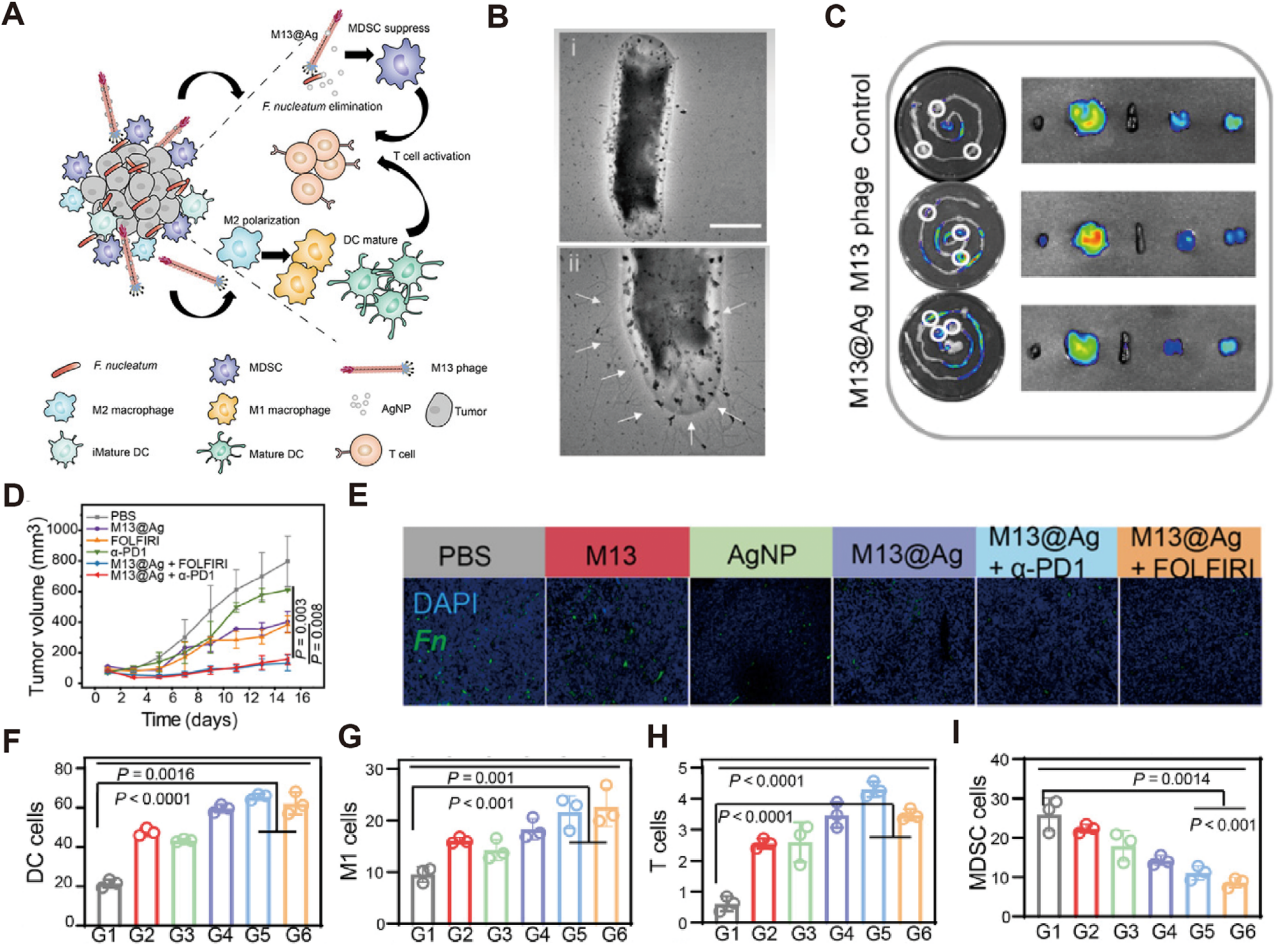
Bioinorganic hybrid bacteriophage for modulation of intestinal microbiota to remodel tumor‐immune microenvironment against colorectal cancer. (A) Schematic illustration of phage‐based bio/abiotic hybrid system (M13@Ag). (B) TEM images of M13@Ag targeting Fn (B‐i and B‐ii). Scale bars= 1 µm. (C) Ex vivo fluorescence imaging of M13 phages and M13@Ag after the intravenous injection at 24 h (*n* = 3). (D) Tumor growth curves. (E) Representative fluorescence images of orthotopic tumor‐bearing mice showing the number of Fn. (F) The mature of CD80^+^CD86^+^ dendritic cell (*n* = 3). (G) TAM of classical activation M1 phenotype highly expressed CD86 (*n* = 3). (H) The level of tumor infiltration CD8^+^T cells (*n* = 3). (I) Percentage of CD11b^+^Gr‐1^+^MDSCs (*n* = 3) [91]. Copyright © 2020 American Association for the Advancement of Science.

Additionally, there is the combination of bacteriophages with inorganic metal antimicrobial nanoparticles to eliminate bacteria in tumors, while also synergistically enhancing immunotherapy and chemotherapy. Xue Dong et al. screened a specifically *Fn*‐binding M13 phage screened by phage display technology. Then, silver nanoparticles (AgNP) were assembled electrostatically on its surface capsid protein (M13@Ag) to achieve specific clearance of *Fn* and remodel the tumor‐immune microenvironment (Figure 7A). M13@Ag could strongly bind to *Fn* (Figure 7B) and effectively target *Fn*‐infiltrated tumor tissues in the orthotopic CT26 murine model (Figure 7C). In vivo, M13@Ag combined with immune checkpoint inhibitors (α‐PD1) or chemotherapeutics (FOLFIRI) significantly inhibited the progression of orthotopic CRC(Figure 7D). The group treated with M13@Ag showed a significant reduction in *Fn* load (Figure 7E). M13 phage could directly stimulate the host immune system, which induced dendritic cell (DC) maturation (Figure 7F) and promoted the activation of M1‐phenotype tumor‐associated macrophages (TAMs) (Figure 7G). Additionally, M13@Ag enhanced the infiltration of CD8^+^ T cells (Figure 7H) and inhibitd the recruitment of MDSC cells (Figure 7I) [91].

### 4.3 Anti‐Tumor Strategy Based on targeting Tumor Sites With Intratumoral Bacteria Characteristics

Tumor cells have an increased demand for oxygen, but inadequate blood vessel density, disrupted vessel structure, or impaired vessel function results in insufficient blood supply to meet the needs of tumor cells, leading to a hypoxic state at the tumor site. This situation is conducive to the colonization and proliferation of many anaerobic bacteria, which can mediate the targeting of various anaerobic bacteria within the tumor [92]. Bacteria's surfaces contain amino groups that can be used for surface modification of nanoparticles by attaching functional groups such as carboxyl or aldehyde groups that can form chemical bonds with the amino groups. This allows for the creation of bacterial/nanoparticle composites via the formation of amide and imine bonds [93]. Therefore, anaerobic bacteria carrying nano drugs can target the interior of tumors through anaerobic targeting and release drugs to kill tumor cells. For example, Cai et al. covalently attached nano photosensitizers (indocyanine green‐loaded nanoparticles [INPs]) to the surface of *Salmonella* *Typhimurium* YB1 via amide bonds to form a biotic/abiotic cross‐linked system (YB1‐INPs) for precise tumor therapy. The composite was injected intravenously into tumor‐bearing mice, and under anaerobic targeting driven by bacteria, the nanoparticles accumulated in the tumor tissue. With two near‐infrared light treatments, ICG heated the tumor site to 63°C, achieving complete clearance of tumor cells and bacteria, demonstrating significant tumor cell–killing effects. This method of combining bacteria with nanoparticles with photothermal properties uses the anaerobic targeting of bacteria to improve the targeted delivery efficiency of nanoparticles, reduce the amount of photothermal agents used, and minimize side effects [94].

*H. pylori* is a common inducer of gastric cancer. Pavimol et al. collected the plasma membrane of gastric epithelial cells (e.g., AGS cells) and wrapped it around a polymer core loaded with antibiotics to create AGS‐NPs. AGS‐NPs simulate natural pathogen–host binding interactions and, after oral administration, preferentially bind to *H. pylori* and release effective antibiotic payloads on‐site to enhance antibacterial efficacy [95].

Bacteria surfaces can express various bacterial proteins to achieve functions such as recognition, movement, protection, and invasion [96]. Bacterial lipoteichoic acid (LTA) is a major component of the cell wall in gram‐positive bacteria. It consists of a glycerol phosphate or ribitol phosphate backbone linked to fatty acids, which in turn are anchored in the cytoplasmic membrane. Lipoteichoic acid plays several important roles in bacterial physiology and host–pathogen interactions [97]. Wenfang Song et al. constructed LTA‐MSNs, mesoporous silica nanoparticles modified with bacterial LTA antibodies, and demonstrated their ability to precisely target bacteria in tumors and deliver anticancer drugs to improve the efficacy of cancer treatment in mice with colon, lung, and breast cancer [98].

**FIGURE 8:**
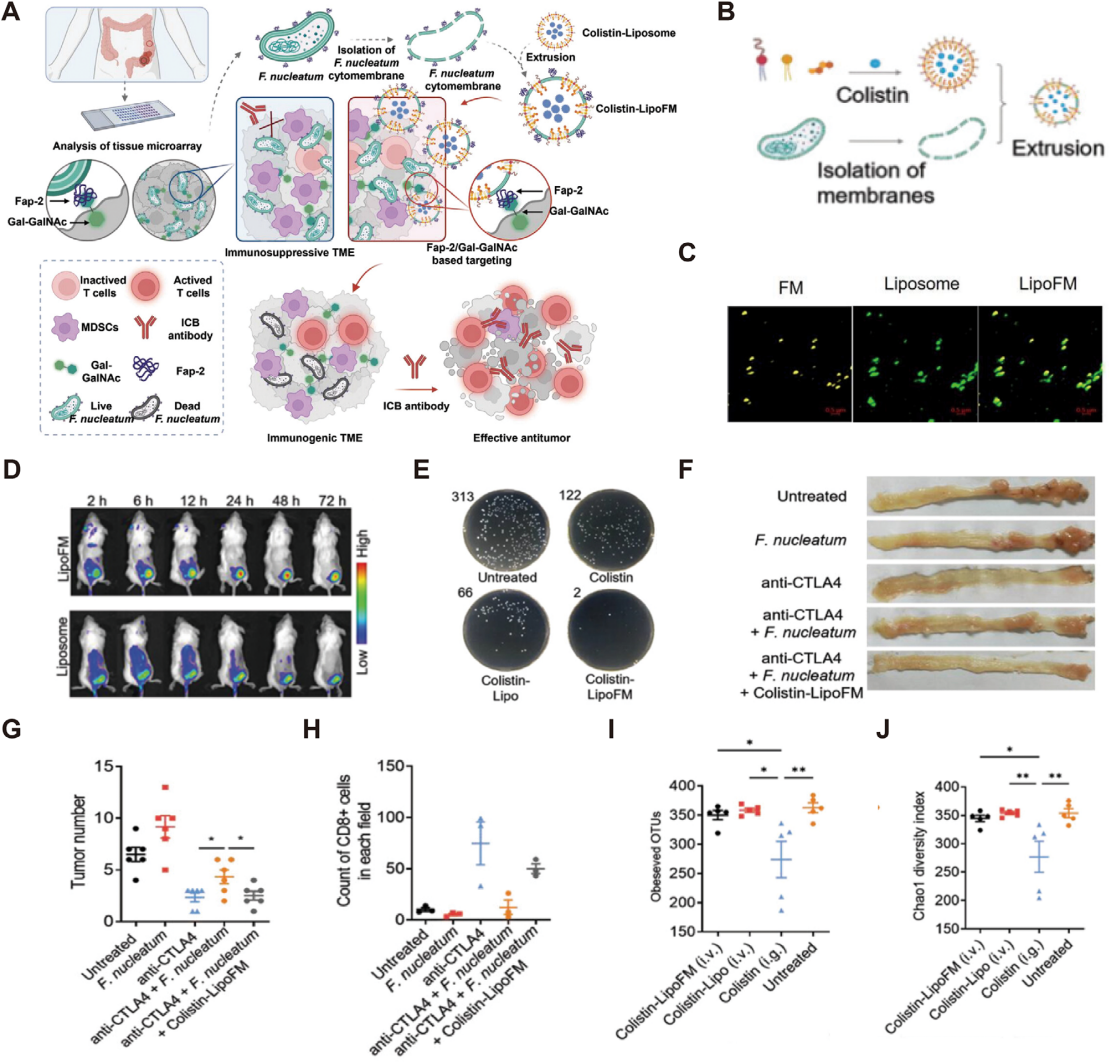
Design and therapy effects of *Fusobacterium nucleatum* mimicking nanomedicine to selectively eliminate tumor colonized bacteria and enhance immunotherapy against CRC. (A) A schematic showing F*. nucleatum* mimicking nanomedicine. (B) Schematic illustrating the preparation of Colistin‐LipoFM. (C) SEM micrographs of Fn treated with PBS (control), MTI, FDU, MTI+FDU, and MTI‐FDU for 12 h. Scale bar = 1 µm. (D) In vivo time‐lapse fluorescence imaging of mice bearing CT26 tumors after intravenous injection with DiR‐labeled liposome or LipoFM. (E) Colony plate images showing the Fn abundance in homogenized tumors of mice with different treatments as indicated. The black number in each picture indicates the number of CFU in each plate. Images (F) and statistical analysis (G) of spontaneous colorectal tumors after different treatments (*n* = 6). (H) Statistical analysis of CD8 +T cells infiltrated in spontaneous colorectal tumors after different treatments (*n* = 3). (I) The estimation of mouse gut microbial community based on observed OTU richness. (J) Chao1 diversity index after different treatments (*n* = 5) [101]. Copyright 2023 Wiley‐VCH GmbH.

It has reported that *F. nucleatum* surface expression of Fap2 can bind to Gal‐GalNac overexpressed on the surface of colon cancer cells [99], allowing it to colonize tumor sites and reduce their response to ICB therapy [100]. Based on this, Linfu Chen et al. designed and constructed a *F. nucleatum*‐mimicking nanomedicine (Colistin‐LipoFM), which selectively killed *F. nucleatum*‐colonizing tumors while successfully restoring responsiveness to ICB therapy (Figure 8A). They fused the cytoplasmic membrane of *F. nucleatum* with a lectin‐loaded liposome to surface express Fap2 (Figure 8B,C). This nanomedicine efficiently targeted tumor sites (Figure 8D) and eliminated *F. nucleatum* (Figure 8E). Meanwhile, Colistin‐LipoFM could enhance the efficacy of anti‐CTLA4 (Figure 8F,G) and effectively reverse the inhibitory effect of *F. nucleatum* on CD8^+^T cell infiltration (Figure 8H). Microbial sequencing of collected fecal samples revealed that Colistin‐LipoFM did not affect the diversity of the gut microbiota (Figure 8I,J) [101].

## 5 Conclusions

With the development of high‐throughput sequencing technology, research on intestinal microbiota has become increasingly mature, and people are gradually realizing the significant role of these tiny organisms in the occurrence and development of tumors within the human body. Consequently, the role of intratumor microbiota in tumor occurrence and cancer treatment has emerged as another intriguing aspect of host–microbiome interaction, worthy of further exploration. Increasing evidence has discovered distinct bacterial characteristics associated with dysbiosis in various types of cancer. Moreover, as research progresses, different bacteria have been found to localize in different parts of the TME, such as the stroma, within tumor cells [74], and within immune cells [102], influencing tumor initiation and progression through various mechanisms. In the pursuit of developing effective antibacterial strategies, nano metallic materials have been found to possess antimicrobial properties [103, 104, 105]. Bacteria present in the TME can influence other components through their own actions, secretion of virulence factors, and metabolites. Harmful bacterial communities complement other components in the immunosuppressive microenvironment and may even form pre‐metastatic niches through hematogenous dissemination, leading to tumor progression, resistance to chemotherapy, radiotherapy, and immunotherapy, resulting in poor prognosis and increased risk of recurrence and metastasis for patients.

Although significant progress has been made in the study of intratumor microbiota, our understanding of it remains limited, mainly due to the low biomass of intratumor microbiota and the limitations of detection technology [106]. For instance, unpredictable environmental or procedural contamination during sample collection, as well as the strong genomic background of the host, pose challenges. These limitations have led to ongoing controversies regarding intratumor microbiota. Consequently, different studies on the same cancer type may yield varying results in bacterial characteristic analyses. Therefore, accurately defining the bacterial characteristics in different tumors remains challenging. Researchers need to adopt various measures to address contamination during sample collection, such as collecting negative controls. Additionally, continuous improvements in decontamination algorithms during sample analysis are essential.

Research on tumor microbiota and its mechanisms in tumor occurrence and development has become one of the emerging directions in anti‐tumor strategies. Traditional antibiotic treatments often lead to various side effects, while the low biomass of intra‐tumoral microbiota demands more precise targeted tumor therapy. Nanotechnology has shown great potential in this regard, and we summarize it into three targeted tumor treatment strategies focusing on intra‐tumoral microbiota.

The strategy of targeting intra‐tumoral microbiota with antibacterial materials combines different types of antibacterial materials (such as antibiotics, natural compounds, and metal ions) with chemotherapy drugs, photosensitizers, and immunotherapeutic drugs to achieve multimodal therapy against tumors. This overcomes the problems associated with high‐dose antibiotics, such as imbalance in intestinal microbiota. However, the drawback is that the antibacterial materials used are often broad‐spectrum and cannot target specific bacteria. The strategy of targeting intra‐tumoral microbiota with bacteriophages addresses this issue by combining bacteriophages targeting specific bacteria with anti‐tumor drugs, enabling precise targeting of tumor sites enriched with those bacteria and efficient tumor killing. However, this strategy is only suitable for bacteria with well‐defined research backgrounds, such as clostridia. The third strategy of targeting intra‐tumoral microbiota fully exploits the characteristics of various types of bacteria. For example, using attenuated anaerobic bacteria as delivery tools can target hypoxic areas of tumors, delivering multiple antibacterial and anti‐tumor drugs, while the delivery tools themselves can also be eliminated. Additionally, targeting the binding sites between bacteria and tumor cells, a nanodelivery system mimicking bacteria can be constructed to achieve precise targeting of tumor sites. Similarly, this strategy is only suitable for well‐defined bacterial species, and attention must be paid to the biological safety of nanodrugs.

Based on the above, we here envision that strategies in nanotechnology targeting intratumoral microbiota in the future may evolve in the following six directions:

1. Nanoparticulation of antibiotics: Nanoparticulating existing antibiotics can enhance their tumor‐targeting ability. Using appropriate doses and combinations of antibiotics can achieve sufficient accumulation at tumor sites to eradicate intratumoral bacteria. This strategy is easily achievable and facilitates clinical translation.
2. Screening of specific narrow‐spectrum antibacterial materials: Apart from antibiotics, certain materials possess antibacterial properties. Nanoparticulating or modifying such antibacterial materials can also achieve tumor‐targeting objectives.
3. Screening of specific bacteriophages: With the gradual elucidation of tumor microbiota profiles, some bacteria with well‐defined roles in promoting cancer have been identified. Developing and screening specific bacteriophages that target these bacteria can eradicate them from tumor sites, thereby alleviating their pro‐cancer effects.
4. Designing antibacterial materials based on bacterial characteristics: Considering the characteristics of certain bacteria colonizing tumor sites, designing nano materials disguised as bacteria can enhance tumor‐targeting capabilities. This approach aims to disrupt bacterial attachment to tumor cells and alleviate their inhibitory effects on immune cells.
5. Combination therapy strategies: Intratumoral bacteria clearance currently serves as an effective adjunct to cancer treatment. Superior antitumor effects can only be achieved by combining this with chemotherapy, targeted therapy, and immunotherapy. Additionally, certain bacteria may exhibit sensitivity to photothermal or SDT, offering antibacterial effects.
6. Precision treatment: Considering the highly heterogeneous nature of intra‐tumoral microbiota, utilizing sequencing techniques such as 16S rRNA or metagenomic sequencing can precisely identify the tumor microbiota of specific populations. Tailoring appropriate antibacterial and antitumor strategies based on specific circumstances may significantly enhance treatment efficacy.

At last, current research on targeting intra‐tumoral microbiota in anti‐tumor studies mostly focuses on well‐defined bacteria such as *F. nucleatum*. Therefore, fundamental research on tumor microbiota is crucial for the development of nano‐delivery vehicles targeting intra‐tumoral microbiota. Similarly, the excellent anti‐tumor effects achieved by nano‐drugs targeting intra‐tumoral microbiota also provide strong support for the study of tumor microbiota. Undoubtedly, the tumor microbiome, as an important component of the TME, is bound to be a hot topic in tumor research and the development of anti‐tumor nano‐drugs!

## Conflicts of Interest

The authors declare no conflicts of interest.
